# Synergetic Impact of Combined 5-Fluorouracil and Rutin on Apoptosis in PC3 Cancer Cells through the Modulation of P53 Gene Expression

**DOI:** 10.15171/apb.2019.055

**Published:** 2019-08-01

**Authors:** Atefeh Satari, Sayed Asadollah Amini, Elham Raeisi, Yves Lemoigne, Esfandiar Heidarian

**Affiliations:** ^1^Clinical Biochemistry Research Center, Basic Health Sciences Institute, Shahrekord University of Medical Sciences, Shahrekord, Iran.; ^2^Cellular and Molecular Research Center, Basic Health Sciences Institute, Shahrekord University of Medical Sciences, Shahrekord, Iran.; ^3^Department of Medical Physics & Radiology, Shahrekord University of Medical Sciences, Shahrekord, Iran.; ^4^Institute for Medical Physics, Ambilly, France.

**Keywords:** Apoptosis, Rutin, 5-Fluorouracil, Prostate Cancer

## Abstract

***Purpose:*** Prostate cancer is as far the most prevalent male cancer. Rutin (a glycoside from quercetin flavonoid) displays antioxidant activity leading to cell apoptosis. Combined effects of rutin with the widely used anti-cancer drug, 5-fluorouracil (5-FU), on prostate cancer cell line (PC3) was investigated herein.

***Methods:*** Different concentrations of combined 5-FU and rutin were applied to PC3 cells compared to separate treatment for 48 hours. Cell viability, as well p53 gene expression respectively were assessed by MTT assay and real-time quantitative polymerase chain reaction (qPCR). Changes of Bcl-2 signal protein and apoptosis were determined using western blot and flow cytometry procedures, respectively. Clonogenic assay was used to colony counts assessment.

***Results:*** 50% inhibitory concentration (IC50) of separate cell treatment with either rutin and 5-FU respectively were 900 μM and 3Mm, while combination index (CI) of combined 5-FU /rutin application reached a level of synergistic effects (0.33). Combination of 5-FU/rutin enhanced apoptosis and p53 gene expression in PC3 cells. PC3 cell colony counts and Bcl-2 signaling protein were decreased by 5-FU/rutin combination.

***Conclusion:*** Synergistic effects of 5-FU/rutin combination on PC3 cells line enhanced apoptosis, p53 gene expression, and down-regulation of Bcl-2 protein, compared to control separate application. 5-FU/rutin combination does seem an interesting therapeutic pathway to be further investigated.

## Introduction

Prostate cancer represents the second widespread cancer and about 10% of all cancers in men.^1^ Although the incidence of prostate cancer in China, Japan, and other Asian countries is lower than the Western countries, it has grown quite rapidly recent years.^2^ Chemotherapy and radiotherapy are the mainstay to treat such a cancer.^3^

5-Fluorouracil (5-FU) is one of the chemotherapy agents widely used as anticancer treatment, especially in the setting of breast and prostate cancers^4^ ; yet, nausea, vomiting, mucositis, stomatitis, and diarrhea remains the major therapeutic side-effects.^5^ Flavonoids are polyphenol compounds which are mainly found in edible and inedible plants with potent antioxidant and anti-radical properties.^6^

Rutin is a glycoside from quercetin flavonoid found in plants such as green tea, and apples.^7^ Rutin has neuroprotection, anti-inflammatory, anti-carcinogenic, antiproliferative, and anti-oxidative stress effects through inhibiting the lipid peroxidation.^8^ Rutin stimulates apoptosis in many cancer cell lines such as prostate and HepG2,^9^ aside reducing Bcl-2 gene and increasing p53 gene expression.^8,10^ P53, a suppressor gene, regulates the cell cycle and acts as a major anticancer barrier.^11^ Bcl-2 proto-oncogene inhibits cell apoptosis and p53 activity.^12^

Chemo-herbal anticancer combination therapy is currently considered as promising. Combining anti-cancer drugs and antioxidant agents do enhance anti-carcinogenic and anti-proliferative effects compared to chemotherapy alone.^13,14^ Rutin and apigenin, as anti-oxidant agents, induce apoptosis in MCF-7 cancer cells through p53-dependent pathway, increasing anti-tumor activity of tamoxifen on cancer cells.^15^

Synergistic effects of anti-oxidant agents and chemotherapeutic drugs (such as combination of gemcitabine, 5-FU, and cisplatin) resort on induction of apoptosis, inhibition of cell proliferation and metastasis invasion.^16-18^ Chemo-herbal combination therapy does attenuate the hazards to inducing drug resistance and prevalence of chemotherapy side effects.^19^ The present study was designed to investigate the synergistic effects of rutin and 5-FU chemo-herbal combination on apoptosis, colony formation, p53 gene expression, and Bcl-2 signaling protein in PC3 prostatic cancer cells.

## Materials and Methods

### Reagents

The human PC3 prostate cancer cells were provided by Pasteur Institute (Tehran, Iran). Trypsin 0.25%, fetal bovine serum (FBS), penicillin/streptomycin (pen/strep), and RPMI 1640 medium were prepared from Gibco (Rockville, MD, USA). 5-FU (50 mg/mL solution) was purchased from Haupt Pharma (Wolfratshausen GmbH Co, Germany). 3-(4,5-dimethylthiazol-2-yl)-2,5-diphenyltetrazolium bromide (MTT) and rutin were obtained from Sigma-Aldrich (St. Louis, MO, USA). Annexin V kit was purchased from BD Bioscience (California, USA). Antibodies were purchased from Elabscience Biotechnology Co. (Wuhan, China). Roti^®^ZOL total RNA extraction kit was prepared from Carl Roth GmbH (Germany). All other chemicals used were of analytical grade.

### Cell viability/proliferation assay

Cell viability was measured by MTT assay test. PC3 cells were seeded in 96-well plates in RPMI 1640 medium (5000 cells/per well) supplement with 10% FBS, 1% pen/strep at 37°C in 98% humidity with 5% CO_2_ for an overnight and then treated with different concentrations of 5-FU (0-10 μM) and rutin (0-1500 μM, solution in DMSO with 0.1% final concentration) for 48 hours. After treatment, medium was removed and the cells were incubated with 10 µL MTT solution (5 mg/mL) for 4 hours at 37ºC in a dark place. Then, DMSO (150 μL) was added to each well in order to dissolve the formazan crystals. Absorbance at 490 nm with a reference wavelength of 570 nm was calculated using a microplate reader (Stat Fax-2100, USA). The percentage of cell viability was assessed based on the absorbance of treated cells as opposed to the untreated control cells (viability = A (sample) / A (control) × 100).^20,21^

### Assessment of synergistic effects of 5-FU and rutin

Synergistic effects assessment of 5-FU and rutin on PC3 cells was based on cell viability. The combined effects of both 5-FU and rutin in different concentrations (0.75 μM 5-FU and 700 μM rutin; 1 μM 5-FU and 500 μM rutin; 1.75 μM 5-FU and 300 μM rutin; and 2.5 μM 5-FU and 100 μM rutin) were assessed for 48 hours. The combination index (CI) was used to evaluate synergistic effects of 5-FU and rutin and CI ˂1, =1 and ˃1 indicated synergism, additive, and antagonism effects respectively.^22^

### Clonogenic assay

PC3 cells were cultured in 6-well plates at density of 2 × 10^2^cells/well for an overnight in RPMI 1640 medium supplement with 10% FBS, 1% pen/strep. Then, PC3 cells were treated with 5-FU (0.75 μM) or rutin (700 μM) separately and using their combination (0.75 μM and 700 μM respectively) for 48 hours. The culture medium was removed and the plates were incubated for 14 days in 5% CO_2_ incubator at 37°C and 95% humidity in the absence of 5-FU and rutin treatment for detecting obvious colonies. The culture medium was changed every 2 days. Then the plates were rinsed with PBS and fixed with 70% ethanol. The staining of colonies was done with a mixture of 0.5% crystal violet in 50:50 methanol:water for 30 minutes. The plates were rinsed with water and left for drying at room temperature. Then, ImageJ software (web-based open source software) was used to evaluate the colonies. The plating efficiency (PE) was measured using the following formula: number of colonies/number of seeded cells ×100 and surviving fraction (SF) was determined by (number of colonies/number of seeded cells × PE control) ×100.^23,24^

### Determining apoptosis

PC3 cells (2×10^5^ per well) were seeded into a 6-well plate in RPMI 1640 medium supplement with 10% FBS, 1% pen/strep and incubated overnight in 5% CO_2_ incubator at 37°C and 95% humidity. Then, the cells were treated with 5-FU (0.75 μM) and rutin (700 μM) or combination of 5-FU and rutin (0.75 μM and 700 μM respectively) for 48 hours. Subsequently, the cells were collected by trypsinization and washed with PBS and stained by Annexin V/propidium (BD Biosciences) based on the manufacturer’s instructions for 25 minutes at room temperature in a dark place.^25^ The stained PC3 cells were analyzed using a flow cytometer (FACScan; Becton Dickinson Immunocytometry Systems, San Jose, CA, USA). Experiments were performed in triplicates.

### Real-time quantitative polymerase chain reaction

PC3 cells were collected after treatment with 5-FU (0.75 μM) and rutin (700 μM) or a combination of 5-FU and rutin (0.75 μM and 700 μM, respectively) in 6-cm dishes after 48 hours. Then, total mRNA was isolated using Roti^®^ZOL reagent according to the manufacturer’s protocol and the RNA quantity and quality were measured by 260/280 nm absorbance ratio using NanoDrop spectrophotometer (Thermo, USA). mRNA was subsequently reverse-transcribed to cDNA using synthesis kit (Takara Bio Inc., Japan). cDNA was amplified by RT-qPCR using SYBR^®^ Green PCR Master Mix (Takara Bio Inc, Japan) in the presence of specific primers for p53 (forward: 5’-CCCATCCTCACCATCATCACAC-3’, reverse: 5’-GCACAAACACGCACCTCAAAG3’), and *GAPDH* (forward: 5′ACACCCACTCCTCCACCCTTTG3′; reverse: 5′GTCCACCACCCTGTTGCTGTA-3′). The primers were designed with Oligo 6.0 (Molecular Biology Insights, Cascade, CO, USA) and confirmed by the blast (NCBI). The primers were obtained from (Macrogen Company, South Korea). The expression of p53 gene was done using Rotor-Gene 3000 (Corbett, Australia). RT-qPCR program consisted of an initial denaturation stage of 95°C at 10 minutes. Then, a three-step program was developed for 40 cycles including 95°C for 10 seconds, 62°C for 15 seconds, and 72°C for 20 seconds, respectively. GAPDH (glyceraldehyde-3-phosphatedehydrogenase) a housekeeping gene, was used as an endogenous control gene for the normalization of p53 expression. The relative quantity of the target gene was determined using the 2^-RΔΔCt^ method.^26^

### Western blot

PC3 cells were plated into 6 dishes (6× 10^5^ cells/dish) in RPMI 1640 medium supplement with 10% FBS, 1% pen/strep and incubated overnight in 5% CO_2_ incubator at 37°C and 95% humidity. The cells treated with 5-FU (0.75 µM), rutin (700 µM), and the combination of 5-FU with rutin (0.75 µM and 700 µM respectively) for 48 hours. Then, the cells were lysed on ice using RIPA buffer (50 mM Tris-HCl with pH 8, 150 mM NaCl, 1% v/v Triton 100X, 0.5% w/v sodium deoxycholate, 1 mM EDTA, 0.1% w/v sodium azide, 50 mM NaF, 0.1% sodium dodecyl sulfate, 1 mM phenylmethylsulfonyl fluoride, proteas and phosphatase inhibitor)^27^ and protein concentrations were measured by Bradford reagent.^28^ The protein samples were mixed with an equal volume of loading buffer (0.125 mM Tris-HCl with pH 6.8, 4% sodium dodecyl sulfate, 20% glycine, and 10% 2-mercaptoethanol) and they were boiled for 5 minutes at 98°C. Denatured proteins were separated by 10% sodium dodecyl sulfate-polyacrylamide gel electrophoresis and were transferred to polyvinylidene difluoride membrane. Then membranes were placed in a blocking solution with 5% BSA for 1 hours. The membranes were washed 3 times in TBS-Tween buffer (containing 10 mM Tris with pH 7.4, 100 mM NaCl, and 0.1 mM Tween-20) for 10 minutes, and they were incubated with primary Bcl-2 and ß-actin (as an internal control) antibodies according to the manufacturer’s protocols at 4°C overnight. Then the membranes were washed with TBS-Tween buffer 3 times for 10 minutes and they were incubated with secondary antibody at room temperature for 2 hours. After washing the membrane 3 times for 10 minutes in TBS-Tween buffer, bands were revealed by enhanced chemiluminescence (ECL; Thermo Fisher scientific, USA).^29^

### Statistical analysis

All data were presented as mean ± standard deviation (SD) in triplicate experiments. Statistical analysis of the data was done using SPSS software version 20 (SPSS Inc., Chicago, IL, USA) and GraphPad Prism 6 (GraphPad software, San Diego, CA). Kruskal-Wallis and Dunn’s test were used to estimate the statistical analysis between the treated and the control groups for real-time PCR, Annexin V assay, clonogenic assay, and MTT assay. The p value less than 0.05 was considered significant. For gene expression analysis, the data were normalized to GAPDH and results were expressed as fold change. The relative levels of quantitative gene expression were estimated with 2^-R∆∆CT^ method. A melting curve analysis of each product was generated to ensure the purity of the amplification product of each reaction. Combination index (CI) was calculated using CompuSyn software (Combo SynInc, City, State, USA) and CI ˂1, =1 and ˃1 were considered as synergism, additive, and antagonism effects respectively.

## Results and Discussion

### Effect of 5-FU and rutin on PC3 cell proliferation inhibition

**Figure 1:**
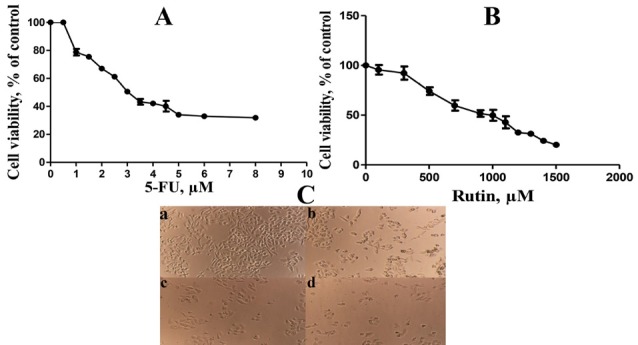
Anti-proliferation effect of **(A)** 5-FU treatment, **(B)** rutin treatment, and **(C)** morphological changes of PC3 cells after treatment with various concentrations of 5-FU with rutin and combination both after 48 h. **a:** (control), **b:** (5-FU, 0.75µM), **c:** (rutin, 700 µM), and **d:** 5-FU with rutin (0.75 µM and 700 µM respectively). Data are expressed as mean ± SD of 3 independent experiments.

Figure 1A and B show cell viability of the PC3 cells treated with different concentrations of 5-FU and rutin for 48 hours. Also, treated PC3 cells showed morphological changes in comparison with the control cells (Figure 1C). IC_50_ values of 5-FU and rutin were 3 μM and 900 μM respectively paralleling previous reports.^30^ Combination of 5-FU and rutin on the PC3 cells viability showed a synergistic effect, by displaying CI reaching 0.33 (Table 1). Cell viability by the current chemo-herbal combination was less than 30%, in agreement with previous studies.^30^ It was shown that CI less than 1 and approaching zero leads to less than 50% cell viability^30^ as observed by the current study.

**Table 1:** The viability percentage of PC3 cells after treated with combination of rutin and 5-FU

**Combination number**	**Dose combination, µM**		**Cell viability, %**	**CI**
	**Rutin (IC value)**	**5-FU (IC value)**		
No. 1	100(IC_10_)	2.5 (IC_40_)	57.80±2.75	1.17
No. 2	300 (IC_20_)	1.75 (IC_30_)	60.26±2.48	1.16
No. 3	500 (IC_30_)	1 (IC_20_)	63.72±3.87	1.27
No. 4	700 (IC_40_)	0.75(IC_10_)	28.03±2.99	0.33

The results were expressed as mean ± SD of three separate experiments.

Natural antioxidant agents and flavonoids are easily accessible through the nature, and taking advantage of their additive cytotoxic efficiency to chemotherapeutic regimens is an ongoing field of investigation to increase expression of anti-cancer genes; yet, limiting chemotherapeutic side effects.^31,32^ Antioxidant agents protect cells against superoxide and hydroxyl free radicals through reported direct and indirect pathways.^17,33^ Since 5-FU has toxic properties and clinical side effects; therefore, attempts were directed to combine 5-FU with other antioxidant agents that does reduce the therapeutic dose of 5-FU, while delaying development of drug resistance. The latter is reported to be sustained by multiple targeting mechanisms to chemo-herbal therapeutic anti-cancer combination.^18,34^ Rutin and 5-FU chemo-herbal combination investigated by the current study displayed superior impact on PC3 cell proliferation’s reduction to sole application of 5-FU or rutin as observed by Wang et al.^30^

The ability to reduce cancer cell proliferation using diverse chemo-herbal combination does effect through activation of apoptosis, caspases 3, 8, 9, down-regulation related to genes deemed as Bcl-2, Bcl-XL, XIAP, and potentiate mediators’ expression.^35-37^ , 5-FU/ rutin chemo-herbal combination used in the current study led to superior reduction in cell proliferation; though reducing 5-FU doses (Table 1 and Figure 1).

### Effect of 5-FU and rutin on apoptosis, gene expression of p53 and cellular pathway Bcl-2 in PC3 cell

**Figure 2:**
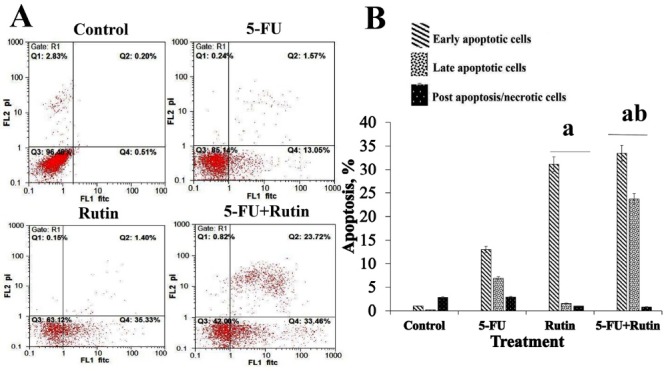
Effect of 5-FU and rutin on the apoptosis of PC3 cells. **(A)** Dot-plots from flow cytometric illustrating apoptotic status in PC3 cells. **(B)** Total percentage of apoptosis in PC3 cells treated with the indicated concentrations of 5-FU (0.75 µM) and rutin (700 µM) or a combination of 5-FU with rutin (0.75 µM and 700 µM respectively) for 48 h. All data were expressed as the mean ± standard deviation. ^a^*P* < 0.05 vs. control cells. ^b^*P* < 0.05 vs. 5-FU treated cells.

**Figure 3:**
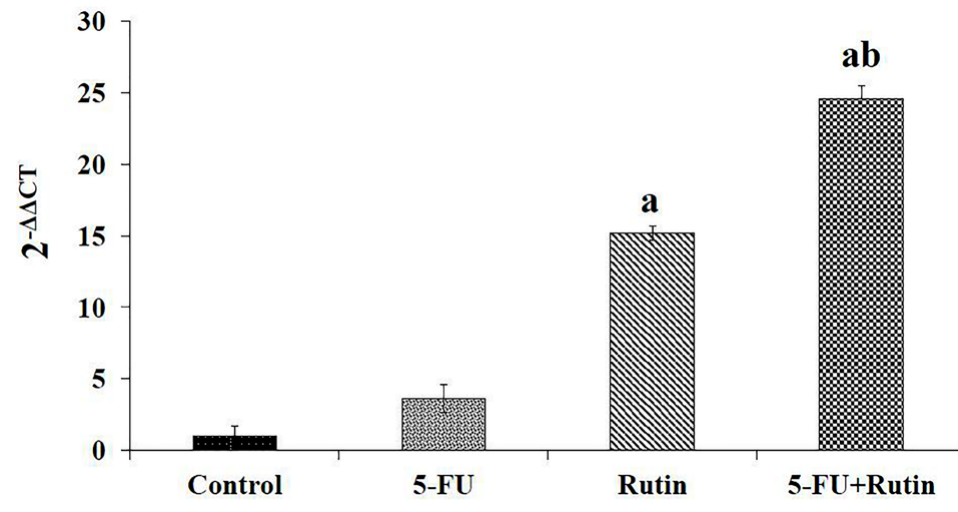
Expression of p53 in PC3 cells at 0.75µM of 5-FU and 700 µM rutin or a combination of 5-FU with rutin (0.75 µM and 700 µM respectively) for 48 h. The expression of p53 was normalized to *GAPDH*. Columns and bars demonstrated mean ± SD of three separate experiments. ^a^*P *< 0.05 *vs.* control cells. ^b^*P *< 0.05 vs. 5-FU treated cells.

**Figure 4:**
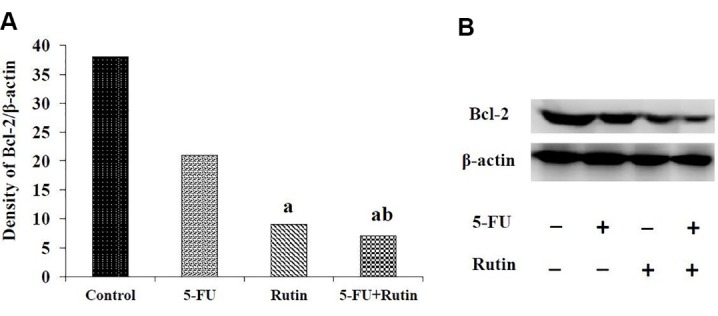
(A) Quantification of Western blot bands by densitometry. **(B)** Western blot analysis performed on PC3 cells were treated with 5-FU (0.75 µM), rutin (700 µM), and combined doses of 5-FU (0.75µM) and rutin (700 µM) for 48 h. a^a^*P* < 0.05 *vs.* control cells. b^b^*P* < 0.05 vs. 5-FU treated cells.

Figures 2, 3 and 4 display the observed effects of 5-FU/rutin combination on apoptosis, p53 gene expression and Bcl-2 cellular signaling protein. The PC3 cells apoptosis percentages using separate or combination of 5-FU and rutin application, respectively were 15.2%, 36.73%, and 57.8%. Comparing to the separate or control groups, 5-FU/rutin combination significantly (*P* < 0.05) increased apoptosis (Figure 2). Rutin applied separately or in combination to 5-FU (FFigure 3) led to enhance p53 gene expression in PC3 cell line by 15.2, and 24.6 fold than control cells (*P* < 0.05). 5-FU/rutin combination displayed superior level of Bcl-2 protein suppression in PC3 cells (Figure 4).

Curcumin in association with 5-FU was reported to stimulate apoptosis, nuclear factor kappa B and p53 gene expression, and reduction to Bcl-2 protein in cancer cells,^38^ paralleling the results of the current study (Figures 2, 3, and 4). As previously reported, 5-FU used separately or in combination with quercetin (a natural antioxidant agent), and melatonin in the setting of human liver and colon cancer cells resulted in a significant cell proliferation inhibition; thereby, empowering apoptosis.^34,39^ 5-FU/rutin combination investigated herein displayed similar finding in respect to cell apoptosis enhancement (Figures 1 and 2). The latter effect on cell apoptosis, reduction in Bcl-2 gene expression and Bcl-2/Bax ratio reported^10,40,41^ with rutin corroborates the current results (Figures 3 and 4).

The anti-proliferative mechanism of antioxidant agents such as rutin was described occurring through prolongation of initial and final apoptosis process by over-expression of Gstp1, Cyp1A1, p38 in special.^7,42^ Anti-cancers chemotherapeutic properties are thought to be empowered using combination with antioxidant agents, in particular by leading towards apoptosis induction.^42,43^ Increasing the Bax and caspase 3, 8, 9 expressions, designed as positive apoptosis regulator, in one hand, and reduction in Bcl-2 expression on human colon cancer cells in another, were found using combination of rutin and hyperoside.^44^ Rutin alone or in combination to 5-FU/oxaliplatin regimen in face of Caco-2 human colon cancer cells, caused phospho-Bad, cleaved caspase 3, and cleaved PARP level up-taking .^31^ Antioxidant agents such as rutin cause the release of cytochrome C from mitochondria into the cytoplasm and activate apoptosis via p53.^45^ The aptitude of antioxidant agents to inhibit cell proliferation and induce caspase-dependent apoptosis is sustained by activating p53 tumor suppresser protein, and down-regulating MDM2.^46^

5-FU has anticancer activities by increasing apoptosis through induction of p53-dependent mitochondrial pathway, releasing cytochrome C from mitochondria into cytoplasm, and activating caspase-9 and caspase3. 5-FU reduces expression of anti-apoptotic protein Bcl-2.^47^ An alternative mitochondrial mechanism leading to potentiate cell apoptosis through expression of p53 (a tumor suppressor gene) that binds to Bcl-2 and Bcl-XL proteins, therefore, activating Bak and Bax apoptotic proteins is advocated.^48^ Therefore, in the present study induction of PC3 cell apoptosis may be, at least partly, due to elevation of p53 gene expression plus reduction of Bcl-2 protein. In addition, our results showed that administration of rutin with 5-FU not only increases the anti-tumor effects of 5-FU but also it reduces 5-FU dose.

### Clonogenic assay

**Figure 5:**
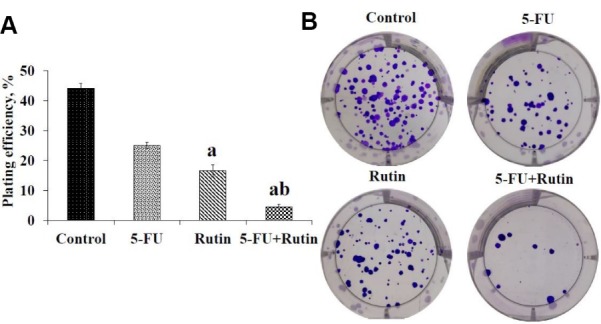
**(A)** Histogram plot demonstrates plating efficiency (PE) in control and treated experimental groups. **(B)** Colony formation images of control, 5-FU, rutin, and 5-FU with rutin combination in PC3 cell. ^a^*P *< 0.05 *vs.* control cells. ^b^*P *< 0.05 *vs.* 5-FU treated cells.

Figure 5 displays the number of colonies in treated PC3 cell in the presence and absence of 5-FU, rutin, and their combination after 14 days. The dishes consisting of 88, 50, 33, and 9 colonies for control, 5-FU, rutin and combination of 5-FU with rutin respectively (Figure 5B). The number of colonies in the setting of 5-FU/rutin combination were remarkably less than that of control or separate application of 5-FU. Surviving fraction (SF) of colonies for 5-FU, rutin, and 5-FU/rutin combination were 56.8%, 37.5%, and 10.2% respectively. Figure 5A  shows PE in control and treated experimental groups. PE showed a significant decrease (*P* < 0.05) in 5-FU/rutin combination in comparison with the control group or separately application of 5-FU. The current results pointed out that the number of colonies and Bcl-2 protein diminished in the case of 5-FU/rutin combination, compared to untreated control cells or by 5-FU application lonely (Figures 5 and 4). Therefore, the reduction in the number of colonies and Bcl-2 signaling protein in 5-FU/rutin combination does reflect the expected synergetic impact on PC3 cancer cells apoptosis induction.

As the main limitation, effects of 5-FU/rutin combination on cell cycle factors such as G2, G1 and NF-Kappa, p38, caspases pathway, and the expression of pro-apoptotic genes (Bax and Bak) were not assessed by the current study. Thus, we suggest that future studies focus on the combined effects of rutin and 5-FU on the above factors.

## Conclusion

The current study reported 5-FU/rutin combination as being prone to significantly inhibit PC3 cell proliferation, inducing apoptosis *via* down-regulation of Bcl-2, and activating the tumor suppressor protein p53 in PC3 human prostatic cancer cells. Nevertheless, further investigations are needed to assess 5-FU/rutin combination on expression of others anti or pro-apoptotic pathways.

## Ethical Issues

The current article does not contain any studies with human or animal subjects.

## Conflict of Interest

The authors declare that there is no conflict of interest.

## Acknowledgments

This study was funded by Research Deputy of Shahrekord University of Medical Sciences (grant no. 2496). We would like to express our gratitude to those who have helped us in Clinical Biochemistry Research Center of Shahrekord University of Medical Sciences. The results described in this paper were the MS dissertation of Miss Atefeh Satari.
